# Defensive Nursing Practices Among Nurses Working in Private Hospitals: A Cross‐Sectional Study From Türkiye

**DOI:** 10.1155/jonm/3933585

**Published:** 2026-11-06

**Authors:** Berna Eren, Fatih Demir

**Affiliations:** ^1^ Department of Healthcare Management Faculty of Health Sciences Acibadem Mehmet Ali Aydinlar University Istanbul Türkiye acibadem.edu.tr; ^2^ Department of Healthcare Management Graduate School of Health Sciences Acibadem Mehmet Ali Aydinlar University Istanbul Türkiye acibadem.edu.tr; ^3^ Operations Directorate Acibadem Mehmet Ali Aydinlar University Istanbul Türkiye acibadem.edu.tr

**Keywords:** defensive medicine, defensive nursing practice, documentation, informed consent, malpractice, nurses, nursing management, patient rights

## Abstract

**Background:**

Defensive medicine affects not only physicians but also other healthcare professionals. Although widely examined among physicians, studies on defensive practices among nurses remain limited. Nurses’ responsibilities in direct care, documentation, communication and informed consent may expose them to malpractice‐related concerns.

**Aim:**

This study aimed to identify defensive nursing practices among nurses working in selected private hospitals in Türkiye and to examine their associated factors.

**Methods:**

A descriptive cross‐sectional survey was conducted with 394 nurses working in selected private hospitals affiliated with four private healthcare groups in Türkiye. Data were collected in April 2023 using an online structured questionnaire covering sociodemographic and professional characteristics, malpractice‐related awareness and 10 defensive nursing practice items. Items were analysed individually rather than as a total score. Descriptive statistics and Pearson’s chi‐square tests were used.

**Results:**

The most frequently reported practices potentially related to defensive nursing were detailed record‐keeping, attention to informed consent forms, detailed explanations and spending more time with patients. Administering unnecessary medications and avoiding patients perceived as legally risky were reported less often. Concern about making mistakes differed by educational level, professional experience, marital status and malpractice‐related awareness variables. Perceived malpractice risk was associated with age, marital status, professional experience and institutional experience. Nurses who believed that malpractice lawsuits affect nursing performance more frequently reported some communication‐ and documentation‐oriented behaviours potentially related to defensive nursing practice.

**Conclusion:**

The reported item‐level responses suggested that practices potentially related to defensive nursing were more commonly reflected in documentation‐, communication‐ and informed consent‐oriented behaviours than in overt avoidance. These findings should be interpreted cautiously, because such behaviours may reflect appropriate professional care when guided by patient safety, patient rights and ethical practice, but may indicate defensive motivation when primarily driven by legal concern or self‐protection. Nurse managers should address malpractice‐related concerns through clear documentation standards, legal and ethical education, communication training and supportive safety cultures.

**Implications for Nursing Management:**

Nurse managers should address defensive nursing practices through clear documentation standards, malpractice‐related education, communication training and supportive safety cultures that help nurses distinguish safe professional caution from fear‐driven self‐protection while strengthening patient‐centred care.

## 1. Introduction

Defensive medicine refers to healthcare practices primarily motivated by the need to avoid malpractice allegations, complaints, disciplinary processes or litigation rather than by the direct clinical benefit of the patient. The term has been used in the medical literature since the late 1960s, and its most common definition refers to physicians’ deviations from sound medical practice due to fear of liability claims or lawsuits [1]. Defensive medicine is generally discussed in two forms: positive or assurance‐based defensive medicine, which includes additional tests, procedures, referrals, documentation or explanations intended to provide legal protection, and negative or avoidance‐based defensive medicine, which involves avoiding high‐risk patients, procedures or clinical responsibilities due to malpractice‐related concerns [1–3]. In nursing, however, defensive practice should be understood within the specific context of nurses’ continuous bedside presence, direct patient care responsibilities, documentation duties, communication with patients and families, and participation in informed consent and care coordination processes [4, 5].

Defensive medicine has been reported across different countries, healthcare systems and specialties, although its prevalence varies widely depending on setting, specialty and measurement approach. Previous reviews have identified assurance and avoidance behaviours as the two major forms of defensive medicine and have shown that defensive medicine is common among physicians, with previous malpractice‐related experience being one of the important determinants [2, 3]. The literature also points to several factors that may contribute to defensive medicine, including fear of litigation, patient complaints, legal uncertainty, workplace violence, media pressure, patient expectations, organisational climate and health policy pressures [6–9].

Within the nursing context, defensive nursing practice may overlap with concepts such as professional caution, risk aversion and conservative clinical decision‐making. This overlap is important because not every cautious or risk‐reducing action should be interpreted as defensive. Careful documentation, detailed explanations, adherence to patient rights procedures and avoidance of unnecessary harm may reflect good professional practice when they are primarily guided by patient safety and ethical care. These behaviours become defensive when the main motivation shifts from patient‐centred care to self‐protection against complaints, disciplinary processes or malpractice allegations. Therefore, distinguishing defensive nursing practice from appropriate professional caution is essential for both research and nursing management [1, 10, 11]. Recent nursing‐specific literature further emphasises that defensive nursing is shaped by risk aversion, organisational expectations, restrictive or overly cautious practices, and the tension between patient safety, autonomy and professional accountability [12–14].

The consequences of defensive medicine extend beyond resource use and cost. Positive defensive practices may contribute to overuse of tests, medications, consultations or documentation, whereas negative defensive practices may restrict access to care for patients perceived as risky or complex [2, 3]. From an ethical and professional perspective, defensive practices may shift the focus of care from the patient’s best interests to the professional’s need for self‐protection, potentially weakening trust and affecting autonomy, beneficence and justice [10]. At the same time, some behaviours that may appear defensive, such as detailed documentation, clear explanation of procedures, careful attention to informed consent and effective communication, may also support patient safety and continuity of care when performed as part of professional standards. Therefore, distinguishing protective professional practice from defensive practice mainly driven by legal fear is important for healthcare management and nursing practice [10, 15].

The Turkish context is also relevant to understanding defensive practices. Developments in patient rights, medical malpractice claims, legal accountability and professional responsibility have contributed to growing interest in defensive medicine in Türkiye [16, 17]. The distinction between malpractice and complication remains a key issue in health law, and uncertainty around this distinction may contribute to defensive behaviours among healthcare professionals [18]. Studies conducted among physicians in Türkiye have reported insufficient knowledge about defensive medicine, concern about malpractice lawsuits and various positive and negative defensive behaviours, including more detailed explanations, more detailed record‐keeping, greater attention to informed consent and avoidance of high‐risk practices [9, 19].

Although defensive medicine has historically been examined mainly in relation to physicians, it is increasingly recognised as a broader healthcare issue that may also concern non‐medical healthcare professionals. A review of defensive practices among non‐medical health professionals showed that defensive behaviours may occur among nurses, midwives and other healthcare professionals, including both active practices such as over‐investigation or over‐treatment and passive practices such as avoiding high‐risk patients [20]. Nurses are central to direct patient care, treatment implementation, documentation, patient education, informed consent processes and communication with patients and families. As nurses’ roles and responsibilities expand, their exposure to malpractice‐related concerns may also increase. Turan and Kaya [4] emphasised that developments in science and technology have changed nurses’ roles and responsibilities and may place nurses in risky circumstances in terms of malpractice. Their study also noted that evidence on whether nurses engage in defensive practices was limited, despite the importance of the issue for nursing care and patient rights. More recently, Ulupınar et al. [5] developed the Defensive Nursing Practices Scale and highlighted the need for a more systematic understanding of defensive behaviours among nurses.

Nurses are particularly important in this context because they are continuously involved in patient monitoring, care implementation, medication administration, documentation, communication and coordination with other healthcare professionals. These responsibilities may expose nurses to malpractice‐related concerns, especially in private hospital settings where patient expectations, service quality standards, documentation requirements and institutional accountability mechanisms are highly visible. For nurse managers, defensive nursing practices are therefore not only individual professional responses but also organisational signals that may reflect perceived legal vulnerability, communication difficulties, workload pressures or uncertainty about institutional support [4, 5, 21].

Existing evidence among nurses suggests that defensive practices may appear particularly in the form of concern about making mistakes, detailed explanations, detailed record‐keeping, avoidance of high‐risk situations and attention to legal responsibilities. Turan and Kaya [4] reported that detailed record‐keeping was one of the most prominent defensive practices among nurses, while perceived malpractice risk and concern about nursing practices were also common. In a recent cross‐sectional study from Egypt, Zaher et al. [21] found that positive defensive practices such as detailed documentation and thorough explanation of procedures were highly prevalent among nurses, whereas negative practices such as avoiding high‐risk procedures or patients more likely to file lawsuits were less frequent. These findings suggest that defensive practices in nursing may not be limited to overt avoidance but may also be embedded in routine professional activities such as documentation, communication and consent‐related practices. Nevertheless, empirical evidence on defensive practices among nurses remains limited, particularly in private hospital settings.

Therefore, this study aimed to identify defensive nursing practices among nurses working in selected private hospitals in Türkiye and to examine sociodemographic, professional and malpractice‐related factors associated with these practices. By focusing on nurses’ item‐level responses rather than a total defensive nursing practice score, the study sought to distinguish between different patterns of defensive practice, including protective communication‐ and documentation‐oriented behaviours and more avoidant or ethically problematic behaviours.

## 2. Methods

### 2.1. Study Design

This study was designed as a descriptive cross‐sectional survey to examine defensive nursing practices and their associations with sociodemographic, professional and malpractice‐related awareness factors.

### 2.2. Setting and Participants

The study was conducted among nurses working in selected private hospitals affiliated with four private healthcare groups in Türkiye. A convenience sampling approach was used. The four healthcare groups were selected because institutional contact points could be established and permission or support for questionnaire distribution could be obtained during the study period. The study population consisted of nurses actively working in the participating private hospital settings during the data collection period. Nurses who agreed to participate and completed the online questionnaire were included in the study. No minimum duration of professional or institutional experience was required, because the aim was to capture defensive nursing practice perceptions among nurses currently working in private hospital settings, including newly employed nurses who may also encounter malpractice‐related concerns during clinical practice.

Questionnaire distribution was facilitated through nursing managers/directors, hospital‐level managers or institutional communication channels. In some groups, distribution was coordinated through hospital‐level contacts, whereas in others it was facilitated through nursing directorate or headquarters‐level communication channels. Because the survey link was disseminated through internal communication networks, including WhatsApp groups, the exact number of hospitals and nurses that received the invitation could not be verified. Therefore, the total eligible population, number of invited nurses and response rate could not be calculated. The final sample consisted of 394 nurses who voluntarily completed the online questionnaire.

### 2.3. Data Collection

Data were collected in April 2023 using an online structured questionnaire created via Google Forms. The questionnaire link was shared with nurses through institutional contact points and internal communication channels. The introductory information text at the beginning of the online questionnaire explained the purpose and academic nature of the study, the voluntary nature of participation, confidentiality of responses, absence of identifying information, and participants’ right to withdraw from the study at any time. Completion of the questionnaire was considered voluntary participation in the study.

### 2.4. Data Collection Instrument

The questionnaire consisted of three sections. The first section included sociodemographic and professional characteristics, including age, sex, marital status, educational level, professional experience, institutional experience, position, working unit and the daily number of patients cared for.

The second section included questions on awareness and experiences related to defensive medicine and malpractice. These items were adapted, with author permission, from the knowledge‐level items reported by Başer et al. [22] in their Turkish Defensive Medicine Behaviour Scale study. They were used to describe nurses’ previous exposure to malpractice‐related issues and their awareness of defensive medicine, rather than to measure defensive behaviour itself. The items addressed previous experience of a malpractice lawsuit, perceived effect of malpractice lawsuits on professional performance, prior awareness of defensive medicine and self‐reported knowledge about its content. Permission to use the relevant questions was obtained from the author.

The third section included 10 defensive nursing practice items. The item content was informed mainly by Turan and Kaya’s [4] study on defensive practices among nurses, which used a structured questionnaire to assess defensive practices at the item level. The items were selected because they reflected behaviours or concerns that may occur in nursing practice in relation to malpractice‐related legal concerns, including concern about making mistakes, detailed explanations, detailed record‐keeping, attention to informed consent, perceived malpractice risk and avoidance‐oriented responses. The wording was adapted to the nursing context by referring to nursing care, nursing practices, patients and malpractice‐related concerns in nursing. The items were reviewed for relevance by the researchers and by a nurse with clinical experience. However, no formal content validity index, cognitive interviewing or pilot testing was conducted. Therefore, the items were not treated as a validated psychometric scale, and no total defensive nursing practice score was calculated. Each item was analysed separately as an item‐level indicator of a specific behaviour, perception or concern related to possible defensive nursing practice.

Importantly, the questionnaire items did not directly measure the underlying motivation for each reported behaviour. Therefore, behaviours such as providing more detailed explanations, keeping more detailed records or paying greater attention to informed consent forms were not interpreted as definitive evidence of inappropriate defensive behaviour. Instead, they were interpreted cautiously as behaviours that may reflect defensive motivation when they are primarily driven by legal concern or self‐protection, but may also represent appropriate professional practice when guided by patient safety, patient rights and ethical care.

### 2.5. Defensive Nursing Practice Items

The section on possible defensive nursing practices included 10 items rated on a 5‐point Likert‐type response scale: 1 = *never*, 2 = *rarely*, 3 = *sometimes*, 4 = *frequently* and 5 = *always*. The items addressed concern about making mistakes in nursing care, administering unnecessary medications to protect oneself from malpractice claims, avoiding patients who may be more likely to file a lawsuit, explaining nursing practices in more detail, avoiding practices with a high risk of complications, keeping medical records in more detail, perceived risk of facing malpractice allegations under current working conditions, spending more time with patients, paying more attention to informed consent forms and feeling uneasy in nursing practices after the enactment of the Turkish Penal Code.

### 2.6. Statistical Analysis

Data were analysed using IBM SPSS Statistics. Descriptive statistics were used to summarise the study variables. Continuous variables were presented as mean and standard deviation, while categorical variables were presented as frequencies and percentages.

For association analyses, the five‐point responses to each defensive nursing practice item were collapsed into three categories: never/rarely, sometimes and frequently/always. This recoding was performed to improve interpretability and reduce sparse cell counts while preserving the ordinal distinction between low, intermediate and high frequency of each practice. The original five response categories were retained for descriptive reporting in Table 1 to show the full distribution of responses, whereas the recoded three‐category variable was used only for chi‐square association analyses. Associations between categorical participant characteristics, malpractice‐related awareness variables and defensive nursing practice items were examined using Pearson’s chi‐square tests. Expected cell counts were checked for each analysis, and Cramér’s V was calculated to estimate the strength of association. The level of statistical significance was set at *p* < 0.05.

**TABLE 1:** Distribution and mean scores of defensive nursing practice items.

Defensive nursing practice item	Never *n* (%)	Rarely *n* (%)	Sometimes *n* (%)	Frequently *n* (%)	Always *n* (%)	Mean ± SD
I am concerned about making mistakes in nursing care.	42 (10.7)	78 (19.8)	92 (23.4)	49 (12.4)	133 (33.8)	3.39 ± 1.40
I administer medications that I think are unnecessary to protect myself from claims of medical malpractice.	307 (77.9)	27 (6.9)	33 (8.4)	12 (3.0)	15 (3.8)	1.48 ± 1.03
I avoid patients who are more likely to file a lawsuit related to nursing practices.	282 (71.6)	60 (15.2)	22 (5.6)	12 (3.0)	18 (4.6)	1.54 ± 1.05
I explain nursing practices to patients in more detail.	9 (2.3)	12 (3.0)	19 (4.8)	106 (26.9)	248 (62.9)	4.45 ± 0.90
I avoid practices with a high risk of complications.	191 (48.5)	78 (19.8)	52 (13.2)	35 (8.9)	38 (9.6)	2.11 ± 1.35
I keep medical records in more detail.	7 (1.8)	5 (1.3)	10 (2.5)	81 (20.6)	291 (73.9)	4.63 ± 0.76
I am at risk of facing a medical malpractice claim under my current working conditions.	37 (9.4)	87 (22.1)	90 (22.8)	59 (15.0)	121 (30.7)	3.36 ± 1.36
I spend more time with patients to protect myself from claims of medical malpractice.	10 (2.5)	19 (4.8)	38 (9.6)	140 (35.5)	187 (47.5)	4.21 ± 0.98
I pay more attention to informed consent forms to protect myself from claims of medical malpractice.	5 (1.3)	10 (2.5)	25 (6.3)	98 (24.9)	256 (65.0)	4.50 ± 0.83
I feel uneasy about my nursing practices after the enactment of the Turkish Penal Code.	141 (35.8)	89 (22.6)	114 (28.9)	31 (7.9)	19 (4.8)	2.23 ± 1.16

*Note:* Responses were rated on a 5‐point Likert‐type scale ranging from 1 = *never* to 5 = *always*. The original five response categories are presented for descriptive purposes. Values are presented as *n* (%) unless otherwise stated.

Abbreviation: SD, standard deviation.

Because multiple item‐level comparisons were performed, the analyses were interpreted as exploratory rather than confirmatory. No formal adjustment for multiple testing was applied, as the aim was to identify potential patterns of association rather than to test a single primary hypothesis. Because the defensive nursing practice items were analysed individually and were not considered components of a validated scale, internal consistency analysis and total score‐based comparisons were not performed.

### 2.7. Use of Artificial Intelligence–Assisted Tools

During manuscript preparation, ChatGPT, an artificial intelligence‐assisted language model developed by OpenAI, was used to support language editing, academic phrasing, structural refinement, consistency checks and the presentation of already completed aggregate results. The tool was not used to generate the research idea, collect data, access the raw dataset, perform statistical analyses or make scientific decisions. No identifiable participant data, raw dataset, confidential institutional information or proprietary data were entered into the tool. All AI‐assisted outputs were critically reviewed, revised and verified by the authors for accuracy, consistency with the study data and appropriateness for the target journal. The authors take full responsibility for the accuracy, integrity and final content of the manuscript.

### 2.8. Ethical Considerations

Ethical approval for the study was obtained from the Acıbadem Mehmet Ali Aydınlar University Medical Research Evaluation Committee (ATADEK) (decision no: 2023‐3/89; date: 24 February 2023). The study was conducted in accordance with ethical principles for research involving human participants.

Before starting the online questionnaire, participants were informed about the purpose and academic nature of the study, the voluntary nature of participation, confidentiality of responses and their right to withdraw from the study at any time. No personally identifying information was collected. Participants indicated their voluntary agreement to take part by completing the online questionnaire.

## 3. Results

### 3.1. Participant Characteristics

A total of 394 nurses were included in the study. Participants had a mean age of 28.64 ± 6.90 years. Most were female, single and graduates of a vocational high school of health. Professional and institutional experiences were most commonly between 1 and 5 years. Most participants worked as nurses, and the most common working units were inpatient, specialised and intensive care units (Table 2).

**TABLE 2:** Sociodemographic and professional characteristics of the nurses.

Characteristics	*n* (%) or mean ± SD
Age group	
≤ 24 years	143 (36.3)
25–29 years	144 (36.5)
≥ 30 years	107 (27.2)
Sex	
Female	349 (88.6)
Male	45 (11.4)
Marital status	
Single	258 (65.5)
Married	136 (34.5)
Educational level	
Vocational high school of health	209 (53.0)
Bachelor’s degree	165 (41.9)
Graduate degree	20 (5.1)
Professional experience	
< 1 year	46 (11.7)
1–5 years	145 (36.8)
6–10 years	100 (25.4)
> 10 years	103 (26.1)
Institutional experience	
< 1 year	136 (34.5)
1–5 years	145 (36.8)
6–10 years	51 (12.9)
> 10 years	62 (15.7)
Position	
Nurse	287 (72.8)
Team leader	36 (9.1)
Charge nurse	71 (18.0)
Working unit	
Inpatient units	102 (25.9)
Outpatient/ambulatory care units	59 (15.0)
Emergency/observation units	29 (7.4)
Intensive care units	90 (22.8)
Specialised units	95 (24.1)
Administrative/education units	19 (4.8)
Daily number of patients cared for	
< 3 patients	120 (30.5)
3–6 patients	144 (36.5)
7–10 patients	40 (10.2)
> 10 patients	90 (22.8)
Age, years, mean ± SD	28.64 ± 6.90

*Note:* Percentages were calculated based on the total sample size, *N* = 394.

Abbreviation: SD, standard deviation.

### 3.2. Awareness and Experiences Related to Defensive Medicine and Malpractice

Less than half of the nurses had previously heard of defensive medicine, and only a small proportion reported sufficient knowledge about its content. Few participants had experienced a malpractice lawsuit during their nursing career. However, most believed that malpractice lawsuits affect nursing performance, had read the Patients’ Rights Regulation and reported that informed consent was obtained for nursing practices in their institution. Only a minority reported knowing the malpractice‐related provisions of the Turkish Penal Code (Table 3).

**TABLE 3:** Awareness and experiences related to defensive medicine and malpractice.

Variable	*n* (%)
Had previously heard of the concept of defensive medicine	
Yes	140 (35.5)
No	254 (64.5)
Reported sufficient knowledge about the content of defensive medicine	
Yes	73 (18.5)
No	321 (81.5)
Had experienced a malpractice lawsuit during their nursing career	
Yes	7 (1.8)
No	387 (98.2)
Believed that malpractice lawsuits affect nursing performance	
Yes	312 (79.2)
No	82 (20.8)
Knew the malpractice‐related provisions of the Turkish Penal Code	
Yes	82 (20.8)
No	312 (79.2)
Had read the Patients’ Rights Regulation	
Yes	320 (81.2)
No	74 (18.8)
Reported that informed consent was obtained for nursing practices in their institution	
Yes	306 (77.7)
No	43 (10.9)
No idea	45 (11.4)

*Note:* Percentages were calculated based on the total sample size, *N* = 394.

### 3.3. Defensive Nursing Practice Items

The highest mean scores were observed for keeping medical records in more detail, paying more attention to informed consent forms, explaining nursing practices in more detail and spending more time with patients. These items also had the highest proportions of ‘frequently’ or ‘always’ responses. By contrast, administering unnecessary medications, avoiding patients perceived as more likely to file a lawsuit and avoiding practices with a high risk of complications had lower mean scores and were more commonly reported as ‘never’ or ‘rarely’. Concern about making mistakes in nursing care and perceived risk of facing a malpractice claim showed intermediate levels, whereas feeling uneasy after the enactment of the Turkish Penal Code was reported less frequently (Table 1).

### 3.4. Factors Associated With Defensive Nursing Practice Items

In the exploratory item‐level analyses, several statistically significant associations were identified between nurses’ sociodemographic and professional characteristics, malpractice‐related awareness variables and individual items related to possible defensive nursing practices. However, the effect sizes were generally small, with Cramér’s V values ranging from 0.118 to 0.216. Concern about making mistakes in nursing care differed significantly by educational level (*χ* ^2^ = 20.640, *p* < 0.001, Cramér’s V = 0.162), professional experience (*χ* ^2^ = 19.103, *p* = 0.004, Cramér’s V = 0.156), marital status (*χ* ^2^ = 8.980, *p* = 0.011, Cramér’s V = 0.151), previous awareness of defensive medicine (*χ* ^2^ = 7.041, *p* = 0.030, Cramér’s V = 0.134), belief that malpractice lawsuits affect nursing performance (*χ* ^2^ = 9.078, *p* = 0.011, Cramér’s V = 0.152) and having read the Patients’ Rights Regulation (*χ* ^2^ = 10.600, *p* = 0.005, Cramér’s V = 0.164). Frequent or always concern was highest among nurses with graduate education and among those with 6–10 years of professional experience. Although concern about making mistakes also differed by marital status, the proportion of frequent or always concern was similar between single and married nurses.

Perceived risk of facing malpractice allegations under current working conditions was significantly associated with age group (*χ* ^2^ = 10.957, *p* = 0.027, Cramér’s V = 0.118), marital status (*χ* ^2^ = 7.973, *p* = 0.019, Cramér’s V = 0.142), professional experience (*χ* ^2^ = 22.871, *p* = 0.001, Cramér’s V = 0.170) and institutional experience (*χ* ^2^ = 16.287, *p* = 0.012, Cramér’s V = 0.144). Frequent or always perceived risk increased across age groups and was highest among nurses aged ≥ 30 years. It was also highest among married nurses, those with > 10 years of professional experience and those with > 10 years of institutional experience.

Some communication‐, documentation‐ and patient rights‐oriented defensive nursing practice items were also associated with participant characteristics or malpractice‐related awareness variables. Providing more detailed explanations of nursing practices was significantly associated with institutional experience (*χ* ^2^ = 15.628, *p* = 0.016, Cramér’s V = 0.141) and belief that malpractice lawsuits affect nursing performance (*χ* ^2^ = 8.293, *p* = 0.016, Cramér’s V = 0.145). Keeping medical records in more detail was significantly associated with belief that malpractice lawsuits affect nursing performance (*χ* ^2^ = 18.442, *p* < 0.001, Cramér’s V = 0.216). Spending more time with patients was significantly associated with previous awareness of defensive medicine (*χ* ^2^ = 7.708, *p* = 0.021, Cramér’s V = 0.140) and belief that malpractice lawsuits affect nursing performance (*χ* ^2^ = 6.027, *p* = 0.049, Cramér’s V = 0.124). Paying more attention to informed consent forms was significantly associated with having read the Patients’ Rights Regulation (*χ* ^2^ = 11.272, *p* = 0.004, Cramér’s V = 0.169).

Avoiding practices with a high risk of complications was significantly associated with previous awareness of defensive medicine (*χ* ^2^ = 7.636, *p* = 0.022, Cramér’s V = 0.139), and this behaviour was more frequently reported among nurses who had not previously heard of defensive medicine. Feeling uneasy in nursing practice after the enactment of the Turkish Penal Code was significantly associated with having read the Patients’ Rights Regulation (*χ* ^2^ = 8.594, *p* = 0.014, Cramér’s V = 0.148) and was more frequent among nurses who had not read the regulation. No statistically significant associations were found between defensive nursing practice items and sex, working unit, position, daily number of patients cared for, self‐reported sufficient knowledge about the content of defensive medicine, knowledge of malpractice‐related provisions of the Turkish Penal Code, or institutional informed consent status. Statistically significant associations are summarised in Table 4, and all examined associations, including non‐significant findings, are presented in Supporting Table S1.

**TABLE 4:** Statistically significant associations with defensive nursing practice items.

Variable	Defensive nursing practice item	*χ* ^2^	df	*p*	Cramér’s V
Age group	Perceived risk of facing a malpractice claim	10.957	4	0.027	0.118
Marital status	Concern about making mistakes in nursing care	8.980	2	0.011	0.151
Marital status	Perceived risk of facing a malpractice claim	7.973	2	0.019	0.142
Educational level	Concern about making mistakes in nursing care	20.640	4	< 0.001	0.162
Professional experience	Concern about making mistakes in nursing care	19.103	6	0.004	0.156
Professional experience	Perceived risk of facing a malpractice claim	22.871	6	0.001	0.170
Institutional experience	Explaining nursing practices in more detail	15.628	6	0.016	0.141
Institutional experience	Perceived risk of facing a malpractice claim	16.287	6	0.012	0.144
Previous awareness of defensive medicine	Concern about making mistakes in nursing care	7.041	2	0.030	0.134
Previous awareness of defensive medicine	Avoiding practices with a high risk of complications	7.636	2	0.022	0.139
Previous awareness of defensive medicine	Spending more time with patients	7.708	2	0.021	0.140
Belief that malpractice lawsuits affect nursing performance	Concern about making mistakes in nursing care	9.078	2	0.011	0.152
Belief that malpractice lawsuits affect nursing performance	Explaining nursing practices in more detail	8.293	2	0.016	0.145
Belief that malpractice lawsuits affect nursing performance	Keeping medical records in more detail	18.442	2	< 0.001	0.216
Belief that malpractice lawsuits affect nursing performance	Spending more time with patients	6.027	2	0.049	0.124
Having read the Patients’ Rights Regulation	Concern about making mistakes in nursing care	10.600	2	0.005	0.164
Having read the Patients’ Rights Regulation	Paying more attention to informed consent forms	11.272	2	0.004	0.169
Having read the Patients’ Rights Regulation	Feeling uneasy in nursing practice after the enactment of the Turkish Penal Code	8.594	2	0.014	0.148

*Note:* Pearson’s chi‐square test was used to examine associations between categorical variables. Five‐point Likert‐type responses for defensive nursing practice items were collapsed into three categories for these analyses: never/rarely, sometimes and frequently/always. *χ* ^2^, chi‐square statistic; *p*, probability value; Cramér’s V, effect size for the strength of association. Only statistically significant associations are presented in this table.

Abbreviation: df, degrees of freedom.

## 4. Discussion

This study examined defensive nursing practices among nurses working in selected private hospital settings in Türkiye and identified their associations with sociodemographic and professional characteristics of nurses as well as their malpractice‐related awareness. The findings showed that nurses frequently reported communication‐ and documentation‐oriented behaviours that may be related to possible defensive nursing practice, particularly keeping more detailed medical records, paying greater attention to informed consent forms, explaining nursing practices more thoroughly and spending more time with patients. In contrast, avoidant or ethically problematic practices, such as administering unnecessary medications or avoiding patients perceived as more likely to file a lawsuit, were reported less frequently. This pattern suggests that behaviours potentially related to defensive nursing may be embedded in routine care processes, particularly documentation and communication, rather than being limited to overt avoidance. This interpretation is consistent with recent nursing literature and with the emerging view that defensive practice is also relevant to non‐medical healthcare professionals [5, 12–14, 20, 21].

The prominence of detailed record‐keeping is consistent with previous research on defensive practices among nurses and physicians. Turan and Kaya [4] reported thorough record‐keeping as one of the most common defensive practices among nurses, with 65.5% of participants stating that they always documented care more extensively to protect themselves from malpractice allegations. In the present study, this tendency was even more pronounced, as 94.4% of nurses reported keeping more detailed medical records frequently or always. Similarly, Zaher et al. [21] found that detailed documentation was the most prevalent positive defensive practice among nurses in Egypt. Physician studies in Türkiye have also identified detailed record‐keeping as a common positive defensive practice [23, 24]. Documentation is a core professional and legal responsibility in nursing, and appropriate record‐keeping supports continuity of care, communication within the healthcare team and patient safety. However, when documentation is primarily driven by fear of blame or litigation, it may also reflect a defensive organisational climate and may turn into a legally oriented task rather than a practice that directly improves care quality [11, 25].

Similarly, high rates of paying attention to informed consent forms and explaining nursing practices in more detail indicate that nurses may respond to malpractice‐related concerns through communication‐based protective behaviours. This pattern is broadly consistent with studies among physicians in Türkiye, where detailed explanations, detailed records and informed consent practices have been reported as common positive defensive practices [19, 24]. It is also consistent with recent nursing evidence showing that thorough explanations of procedures may be among the common positive defensive practices reported by nurses [21]. These behaviours should not be interpreted only as defensive in the nursing context. Clear explanations, informed consent and patient‐centred communication are also essential components of patient rights, autonomy and safe care. The broader literature also suggests that trust, communication and shared decision‐making are important in reducing defensive practice and malpractice‐related tension [26]. Nevertheless, the motivation underlying these practices is important. If such practices are performed mainly to protect professionals rather than to strengthen patient understanding and shared decision‐making, they may represent a defensive rather than patient‐centred orientation.

Compared with the high levels of documentation‐ and communication‐oriented behaviours, nurses in this study reported low levels of administering medications considered unnecessary and avoiding patients perceived as legally risky. This finding is important because these behaviours raise more direct ethical concerns. Administering unnecessary medications may expose patients to avoidable harm and conflict with safe medication practice, while avoiding patients because of anticipated legal risk may compromise equity and access to care. Turan and Kaya [4] similarly found that most nurses did not administer unnecessary drugs and did not avoid patients considered likely to file a lawsuit. Zaher et al. [21] also reported that negative defensive practices, including avoiding high‐risk procedures or patients associated with potential legal claims, were less frequent than positive defensive practices among nurses. By contrast, physician studies have often reported higher levels of avoidant or intervention‐related defensive behaviours, including unnecessary referrals, tests, procedures or avoidance of high‐risk patients and procedures [3, 27]. This difference may partly reflect nurses’ professional role, especially their limited prescribing authority and closer involvement in continuous bedside care. The present study therefore supports the view that nurses’ defensive practices may be more likely to emerge as assurance‐oriented behaviours rather than as overtly avoidant behaviours. This distinction is important for nursing management because interventions should not merely discourage ‘defensive practice’ in general, but should help differentiate appropriate professional caution from legally motivated, unnecessary or ethically problematic behaviour.

Concern about making mistakes in nursing care was reported frequently or always by nearly half of the nurses. This finding is consistent with the notion that malpractice‐related anxiety, uncertainty and concern about medical error are major drivers of defensive practice. Studies among physicians have shown that malpractice fear, previous litigation experience, perceived legal vulnerability and uncertainty in clinical decision‐making are associated with defensive behaviours [3, 9, 28]. Although most of this evidence comes from physicians, the mechanism may also be relevant for nurses, who are directly involved in medication administration, patient monitoring, documentation and bedside care. In the present study, concern about making mistakes was significantly associated with educational level, professional experience, marital status, prior awareness of defensive medicine, belief that malpractice lawsuits affect nursing performance and having read the Patients’ Rights Regulation. These findings indicate that concern about error is shaped not only by individual characteristics but also by awareness of legal and professional responsibilities.

Perceived risk of facing malpractice allegations under current working conditions was also an important finding. This perception was significantly associated with age, marital status, professional experience and institutional experience. Frequent or always perceived risk was highest among nurses aged 30 years or older and among those with more than 10 years of professional and institutional experience. This pattern suggests that more experienced nurses may be more aware of malpractice‐related risks because of longer exposure to clinical incidents, patient complaints, institutional procedures and legal discussions. Turan and Kaya [4] similarly reported that nurses’ concern about making mistakes increased with professional experience and that more experienced nurses were more likely to avoid high‐complication practices. Studies among physicians have also shown that previous malpractice‐related experiences, workplace disputes or longer professional exposure may be associated with defensive practices and behaviours [3, 29, 30]. However, the relationship between experience and defensive practice is not necessarily linear across all studies. Some recent physician studies have suggested that younger or less experienced clinicians may report higher malpractice fear or defensive practice tendencies [9, 24]. These differences may reflect variations in profession, clinical setting, measurement approach and local malpractice‐related conditions.

The association between malpractice‐related performance concerns and protective defensive practices was one of the strongest findings of this study. Nurses who believed that malpractice lawsuits affect nursing performance more frequently reported concern about making mistakes and were more likely to provide thorough explanations, keep detailed records and spend more time with patients. This suggests that perceived performance pressure related to malpractice may be associated with more intensive communication, documentation and time allocation behaviours. From a management perspective, this finding is particularly relevant. If malpractice concerns affect perceived performance, nurses may experience not only legal anxiety but also professional insecurity and increased emotional burden. Behaviours potentially related to defensive nursing may therefore be understood partly as coping strategies within a perceived risk environment, as previous literature has also linked defensive practice to fear of litigation, complaints, uncertainty and organisational pressures [6, 11]. Organisational responses should include supportive supervision, clear documentation standards, legal and ethical education, and supportive and learning‐oriented approaches to error reporting [6, 11, 20].

Having read the Patients’ Rights Regulation was significantly associated with paying more attention to informed consent forms, concern about making mistakes and feeling uneasy after the enactment of the Turkish Penal Code. The strongest and most clinically coherent association was between reading the regulation and attention to informed consent forms. This finding suggests that patient rights awareness may be related to greater sensitivity to informed consent processes. In Türkiye, patient rights, malpractice claims and legal accountability have become increasingly visible in healthcare practice [17, 18]. For nurses, this visibility may strengthen awareness of professional responsibilities, but it may also increase anxiety if not supported by adequate institutional education and guidance. Similarly, awareness of applicable legal responsibilities under the Turkish Penal Code remains relevant for nurses regardless of age or professional experience, although individual interpretations of this item may vary depending on when participants entered professional practice. Therefore, patient rights education should not be limited to legal obligations; it should also clarify how nurses can protect patient autonomy, communicate effectively and document care appropriately without adopting unnecessarily defensive practices.

The findings have several implications for nursing management. First, managers should recognise that defensive practices among nurses may be embedded in routine practices such as record‐keeping, explanations, informed consent and time spent with patients. Because the present questionnaire did not directly assess defensive motivation, these behaviours should be interpreted as possible indicators of defensive nursing practice rather than as definitive evidence of inappropriate or unnecessary care. Second, institutions should provide clear, standardised and practical guidance on documentation, informed consent and patient communication. Third, education on malpractice, patient rights and professional responsibility should be integrated with patient safety and ethical decision‐making, rather than delivered only as legal risk avoidance training. Fourth, supportive organisational climates are needed to reduce fear‐based practice. Nurses should feel that reporting errors, near misses or uncertainty will lead to learning and system improvement rather than blame. This is important because defensive practice is not only an individual behaviour but also a response to organisational, legal and social pressures, including complaint processes, workplace violence, policy pressures, uncertainty in clinical practice and perceived lack of institutional support [6, 8, 10, 11, 20]. Therefore, nursing management strategies should focus not only on reducing fear of litigation but also on creating supportive clinical environments in which nurses can practise safely, communicate openly and distinguish professional caution from fear‐driven self‐protection [11, 26]. This is consistent with recent recommendations that legal literacy, supportive supervision, compassionate leadership and learning‐oriented organisational cultures are needed to help nurses manage legal risk without replacing patient‐centred professional judgement with fear‐driven defensive practice [12, 14].

This study has several limitations. First, the cross‐sectional design does not allow causal interpretations. Therefore, the findings should be interpreted as associations rather than causal relationships. Second, the data were based on self‐reported responses, which may be affected by recall bias and social desirability bias, particularly for ethically sensitive behaviours such as administering unnecessary medications or avoiding patients perceived as more likely to file a lawsuit. Third, the study used a convenience sample of nurses from selected private hospital settings affiliated with four private healthcare groups in Türkiye. Because the questionnaire link was disseminated through institutional contact points and internal communication channels, including WhatsApp groups, the exact number of hospitals and nurses that received the invitation could not be verified. Therefore, the total eligible population, number of invited nurses, response rate and the exact institutional and geographic distribution of respondents could not be calculated. This may have introduced selection and non‐response bias, and the findings should not be considered representative of all nurses working in private hospitals in Türkiye.

Fourth, the defensive nursing practice items were developed with reference to previous literature and adapted to the nursing context, but no formal content validity index, cognitive interviewing or pilot testing was conducted. Therefore, the items should be interpreted as item‐level indicators rather than as a validated scale measuring an overall defensive nursing practice construct. Fifth, the five‐point Likert‐type responses were collapsed into three categories for the chi‐square analyses. Although this approach improved interpretability and reduced sparse cell counts, it may have resulted in some loss of information. Multiple item‐level chi‐square tests were performed, which increases the possibility of type I error. No formal adjustment for multiple testing was applied; therefore, statistically significant findings, particularly those with small Cramér’s V values, should be interpreted cautiously and confirmed in future studies. Finally, self‐reported defensive nursing practices may be difficult to distinguish from appropriate professional caution, patient safety‐oriented behaviour and compliance with documentation or informed consent procedures. For example, detailed record‐keeping or more detailed explanations may represent good professional practice when primarily guided by patient safety and ethical care, but may become defensive when mainly motivated by fear of complaints, disciplinary processes or malpractice allegations. This distinction could not be fully assessed using the present questionnaire. Future studies should examine defensive nursing practices in different institutional settings, compare public and private hospitals, use validated nursing‐specific instruments and explore nurses’ motivations through qualitative or mixed‐method designs.

Despite these limitations, the study contributes to the growing but still limited empirical literature on defensive practices among nurses. Recent studies have begun to develop nursing‐specific measurement tools and examine the prevalence and associated factors of defensive nursing practices in different contexts [5, 21]. The present study adds to this emerging literature by analysing item‐level defensive nursing practice indicators among nurses working in private hospital settings in Türkiye. It highlights that defensive nursing practices may be expressed primarily through documentation‐, informed consent‐ and communication‐oriented behaviours rather than through overt avoidance. This distinction is important for nursing management and patient safety, and should be considered in education on nurses’ legal and ethical responsibilities.

## 5. Conclusion

This study showed that, at the item level, nurses working in selected private hospital settings in Türkiye more frequently reported documentation‐, informed consent‐ and communication‐oriented behaviours that may be related to possible defensive nursing practice, whereas avoidant or ethically more problematic behaviours were less frequently reported. Because the items did not directly assess the underlying motivation for each behaviour and were not analysed as a validated total scale, the findings should be interpreted as exploratory indicators rather than as definitive evidence of a coherent defensive nursing practice construct. Malpractice‐related risk perception, concern about making mistakes, patient rights awareness and perceived performance effects of malpractice lawsuits were associated with several defensive nursing practice items; however, these associations generally had small effect sizes and should be interpreted cautiously. These findings suggest that practices potentially related to defensive nursing should be considered in relation to communication, documentation, patient rights and organisational risk management. Nurse managers should support nurses through clear standards, education on legal and ethical responsibilities, supportive safety cultures and patient‐centred communication strategies to reduce fear‐based practice while strengthening safe and accountable care.

## Author Contributions

Berna Eren contributed to the study conception and design, supervision, data interpretation, manuscript drafting and critical revision. Fatih Demir contributed to data collection, data preparation, statistical analysis and manuscript revision.

## Funding

This research received no specific grant from any funding agency in the public, commercial or not‐for‐profit sectors.

## Disclosure

All authors read and approved the final manuscript.

## Ethics Statement

Ethical approval for the study was obtained from the Acıbadem Mehmet Ali Aydınlar University Medical Research Evaluation Committee (ATADEK) (decision no: 2023‐3/89; date: 24 February 2023). Participation was voluntary. Before completing the online questionnaire, participants were informed about the purpose of the study, confidentiality of responses, absence of personally identifying information and their right to withdraw at any time. Completion of the questionnaire was considered voluntary participation.

## Conflicts of Interest

The authors declare no conflicts of interest.

## Supporting Information

Additional supporting information can be found online in the Supporting Information section.

## Supporting information


**Supporting Information 1** Supporting Table S1 presents the full results of the exploratory item‐level association analyses between participant characteristics, malpractice‐related awareness variables and defensive nursing practice items. The table includes Pearson’s chi‐square test results, degrees of freedom, *p* values, Cramér’s V effect sizes and expected cell count information for both statistically significant and non‐significant associations.

**Supporting Information 2** JONM_STROBE_Checklist_Eren_Demir.

## Data Availability

The data that support the findings of this study are available from the corresponding author upon reasonable request.
