# Supplementary material for: Defensive Nursing Practices Among Nurses Working in Private Hospitals: A Cross‐Sectional Study From Türkiye
Source: J Nurs Manag. 2026 Nov 6;2026:3933585. doi: 10.1155/jonm/3933585 (PMC13743797; doi:10.1155/jonm/3933585)
Supplement: Supplementary file 1 [file JONM-2026-3933585-s001.docx]

**Supplementary Table S1. Associations between participant characteristics, malpractice-related awareness variables and defensive nursing practice items**

This supplementary table presents all Pearson’s chi-square analyses for the defensive nursing practice items, including statistically significant and non-significant associations.

| **Factor** | **Defensive nursing practice item** | **χ²** | **df** | **p** | **Cramér’s V** | **Cells with expected count <5, n (%)** | **Minimum expected count** | **Significant** |
| --- | --- | --- | --- | --- | --- | --- | --- | --- |
| Age group | Concern about making mistakes in nursing care | 6.114 | 4 | .191 | .088 | 0 (0.0%) | 24.98 | No |
| Age group | Administering unnecessary medications to protect oneself from malpractice claims | 4.712 | 4 | .318 | .077 | 0 (0.0%) | 7.33 | No |
| Age group | Avoiding patients perceived as more likely to file a lawsuit | 5.270 | 4 | .261 | .082 | 0 (0.0%) | 5.97 | No |
| Age group | Explaining nursing practices in more detail | 1.120 | 4 | .891 | .038 | 0 (0.0%) | 5.16 | No |
| Age group | Avoiding practices with a high risk of complications | 4.927 | 4 | .295 | .079 | 0 (0.0%) | 14.12 | No |
| Age group | Keeping medical records in more detail | 3.013 | 4 | .556 | .062 | 6 (66.7%) | 2.72 | No |
| Age group | Perceived risk of facing a malpractice claim under current working conditions | 10.957 | 4 | **.027** | .118 | 0 (0.0%) | 24.44 | **Yes** |
| Age group | Spending more time with patients to protect oneself from malpractice claims | 3.297 | 4 | .509 | .065 | 0 (0.0%) | 7.88 | No |
| Age group | Paying more attention to informed consent forms | 5.482 | 4 | .241 | .083 | 1 (11.1%) | 4.07 | No |
| Age group | Feeling uneasy in nursing practice after the enactment of the Turkish Penal Code | 8.913 | 4 | .063 | .106 | 0 (0.0%) | 13.58 | No |
| Sex | Concern about making mistakes in nursing care | 1.452 | 2 | .484 | .061 | 0 (0.0%) | 10.51 | No |
| Sex | Administering unnecessary medications to protect oneself from malpractice claims | 2.091 | 2 | .351 | .073 | 2 (33.3%) | 3.08 | No |
| Sex | Avoiding patients perceived as more likely to file a lawsuit | 2.962 | 2 | .227 | .087 | 2 (33.3%) | 2.51 | No |
| Sex | Explaining nursing practices in more detail | .099 | 2 | .952 | .016 | 2 (33.3%) | 2.17 | No |
| Sex | Avoiding practices with a high risk of complications | 5.137 | 2 | .077 | .114 | 0 (0.0%) | 5.94 | No |
| Sex | Keeping medical records in more detail | 1.463 | 2 | .481 | .061 | 2 (33.3%) | 1.14 | No |
| Sex | Perceived risk of facing a malpractice claim under current working conditions | .709 | 2 | .702 | .042 | 0 (0.0%) | 10.28 | No |
| Sex | Spending more time with patients to protect oneself from malpractice claims | 3.383 | 2 | .184 | .093 | 2 (33.3%) | 3.31 | No |
| Sex | Paying more attention to informed consent forms | 2.038 | 2 | .361 | .072 | 2 (33.3%) | 1.71 | No |
| Sex | Feeling uneasy in nursing practice after the enactment of the Turkish Penal Code | 1.248 | 2 | .536 | .056 | 0 (0.0%) | 5.71 | No |
| Marital status | Concern about making mistakes in nursing care | 8.980 | 2 | **.011** | .151 | 0 (0.0%) | 31.76 | **Yes** |
| Marital status | Administering unnecessary medications to protect oneself from malpractice claims | 4.266 | 2 | .118 | .104 | 0 (0.0%) | 9.32 | No |
| Marital status | Avoiding patients perceived as more likely to file a lawsuit | .430 | 2 | .806 | .033 | 0 (0.0%) | 7.59 | No |
| Marital status | Explaining nursing practices in more detail | .094 | 2 | .954 | .015 | 0 (0.0%) | 6.56 | No |
| Marital status | Avoiding practices with a high risk of complications | .116 | 2 | .944 | .017 | 0 (0.0%) | 17.95 | No |
| Marital status | Keeping medical records in more detail | .102 | 2 | .950 | .016 | 2 (33.3%) | 3.45 | No |
| Marital status | Perceived risk of facing a malpractice claim under current working conditions | 7.973 | 2 | **.019** | .142 | 0 (0.0%) | 31.07 | **Yes** |
| Marital status | Spending more time with patients to protect oneself from malpractice claims | .174 | 2 | .916 | .021 | 0 (0.0%) | 10.01 | No |
| Marital status | Paying more attention to informed consent forms | 4.871 | 2 | .088 | .111 | 0 (0.0%) | 5.18 | No |
| Marital status | Feeling uneasy in nursing practice after the enactment of the Turkish Penal Code | 4.606 | 2 | .100 | .108 | 0 (0.0%) | 17.26 | No |
| Educational level | Concern about making mistakes in nursing care | 20.640 | 4 | **<.001** | .162 | 1 (11.1%) | 4.67 | **Yes** |
| Educational level | Administering unnecessary medications to protect oneself from malpractice claims | 2.185 | 4 | .702 | .053 | 2 (22.2%) | 1.37 | No |
| Educational level | Avoiding patients perceived as more likely to file a lawsuit | 1.515 | 4 | .824 | .044 | 2 (22.2%) | 1.12 | No |
| Educational level | Explaining nursing practices in more detail | 2.290 | 4 | .683 | .054 | 2 (22.2%) | .96 | No |
| Educational level | Avoiding practices with a high risk of complications | 3.360 | 4 | .500 | .065 | 2 (22.2%) | 2.64 | No |
| Educational level | Keeping medical records in more detail | 2.196 | 4 | .700 | .053 | 3 (33.3%) | .51 | No |
| Educational level | Perceived risk of facing a malpractice claim under current working conditions | 3.248 | 4 | .517 | .064 | 1 (11.1%) | 4.57 | No |
| Educational level | Spending more time with patients to protect oneself from malpractice claims | 3.215 | 4 | .523 | .064 | 2 (22.2%) | 1.47 | No |
| Educational level | Paying more attention to informed consent forms | 2.032 | 4 | .730 | .051 | 2 (22.2%) | .76 | No |
| Educational level | Feeling uneasy in nursing practice after the enactment of the Turkish Penal Code | 7.535 | 4 | .110 | .098 | 1 (11.1%) | 2.54 | No |
| Professional experience | Concern about making mistakes in nursing care | 19.103 | 6 | **.004** | .156 | 0 (0.0%) | 10.74 | **Yes** |
| Professional experience | Administering unnecessary medications to protect oneself from malpractice claims | 5.159 | 6 | .524 | .081 | 2 (16.7%) | 3.15 | No |
| Professional experience | Avoiding patients perceived as more likely to file a lawsuit | 1.938 | 6 | .925 | .050 | 2 (16.7%) | 2.57 | No |
| Professional experience | Explaining nursing practices in more detail | 7.243 | 6 | .299 | .096 | 4 (33.3%) | 2.22 | No |
| Professional experience | Avoiding practices with a high risk of complications | 8.622 | 6 | .196 | .105 | 0 (0.0%) | 6.07 | No |
| Professional experience | Keeping medical records in more detail | 4.510 | 6 | .608 | .076 | 8 (66.7%) | 1.17 | No |
| Professional experience | Perceived risk of facing a malpractice claim under current working conditions | 22.871 | 6 | **.001** | .170 | 0 (0.0%) | 10.51 | **Yes** |
| Professional experience | Spending more time with patients to protect oneself from malpractice claims | 3.490 | 6 | .745 | .067 | 2 (16.7%) | 3.39 | No |
| Professional experience | Paying more attention to informed consent forms | 10.808 | 6 | .095 | .117 | 4 (33.3%) | 1.75 | No |
| Professional experience | Feeling uneasy in nursing practice after the enactment of the Turkish Penal Code | 11.940 | 6 | .063 | .123 | 0 (0.0%) | 5.84 | No |
| Institutional experience | Concern about making mistakes in nursing care | 5.756 | 6 | .451 | .085 | 0 (0.0%) | 11.91 | No |
| Institutional experience | Administering unnecessary medications to protect oneself from malpractice claims | 2.325 | 6 | .888 | .054 | 3 (25.0%) | 3.49 | No |
| Institutional experience | Avoiding patients perceived as more likely to file a lawsuit | 7.291 | 6 | .295 | .096 | 4 (33.3%) | 2.85 | No |
| Institutional experience | Explaining nursing practices in more detail | 15.628 | 6 | **.016** | .141 | 4 (33.3%) | 2.46 | **Yes** |
| Institutional experience | Avoiding practices with a high risk of complications | 5.621 | 6 | .467 | .084 | 0 (0.0%) | 6.73 | No |
| Institutional experience | Keeping medical records in more detail | 2.266 | 6 | .894 | .054 | 8 (66.7%) | 1.29 | No |
| Institutional experience | Perceived risk of facing a malpractice claim under current working conditions | 16.287 | 6 | **.012** | .144 | 0 (0.0%) | 11.65 | **Yes** |
| Institutional experience | Spending more time with patients to protect oneself from malpractice claims | 6.007 | 6 | .422 | .087 | 3 (25.0%) | 3.75 | No |
| Institutional experience | Paying more attention to informed consent forms | 5.559 | 6 | .474 | .084 | 4 (33.3%) | 1.94 | No |
| Institutional experience | Feeling uneasy in nursing practice after the enactment of the Turkish Penal Code | 10.164 | 6 | .118 | .114 | 0 (0.0%) | 6.47 | No |
| Position | Concern about making mistakes in nursing care | 5.330 | 4 | .255 | .082 | 0 (0.0%) | 8.41 | No |
| Position | Administering unnecessary medications to protect oneself from malpractice claims | 2.662 | 4 | .616 | .058 | 3 (33.3%) | 2.47 | No |
| Position | Avoiding patients perceived as more likely to file a lawsuit | 4.526 | 4 | .339 | .076 | 3 (33.3%) | 2.01 | No |
| Position | Explaining nursing practices in more detail | 1.997 | 4 | .736 | .050 | 4 (44.4%) | 1.74 | No |
| Position | Avoiding practices with a high risk of complications | 4.404 | 4 | .354 | .075 | 1 (11.1%) | 4.75 | No |
| Position | Keeping medical records in more detail | 4.635 | 4 | .327 | .077 | 4 (44.4%) | .91 | No |
| Position | Perceived risk of facing a malpractice claim under current working conditions | 3.419 | 4 | .490 | .066 | 0 (0.0%) | 8.22 | No |
| Position | Spending more time with patients to protect oneself from malpractice claims | 8.443 | 4 | .077 | .104 | 2 (22.2%) | 2.65 | No |
| Position | Paying more attention to informed consent forms | 2.525 | 4 | .640 | .057 | 4 (44.4%) | 1.37 | No |
| Position | Feeling uneasy in nursing practice after the enactment of the Turkish Penal Code | 4.148 | 4 | .386 | .073 | 1 (11.1%) | 4.57 | No |
| Working unit | Concern about making mistakes in nursing care | 5.177 | 10 | .879 | .081 | 1 (5.6%) | 4.44 | No |
| Working unit | Administering unnecessary medications to protect oneself from malpractice claims | 5.952 | 10 | .819 | .087 | 6 (33.3%) | 1.30 | No |
| Working unit | Avoiding patients perceived as more likely to file a lawsuit | 6.588 | 10 | .764 | .091 | 6 (33.3%) | 1.06 | No |
| Working unit | Explaining nursing practices in more detail | 6.905 | 10 | .734 | .094 | 10 (55.6%) | .92 | No |
| Working unit | Avoiding practices with a high risk of complications | 13.544 | 10 | .195 | .131 | 3 (16.7%) | 2.51 | No |
| Working unit | Keeping medical records in more detail | 6.241 | 10 | .795 | .089 | 12 (66.7%) | .48 | No |
| Working unit | Perceived risk of facing a malpractice claim under current working conditions | 10.200 | 10 | .423 | .114 | 1 (5.6%) | 4.34 | No |
| Working unit | Spending more time with patients to protect oneself from malpractice claims | 6.625 | 10 | .760 | .092 | 5 (27.8%) | 1.40 | No |
| Working unit | Paying more attention to informed consent forms | 3.810 | 10 | .956 | .070 | 9 (50.0%) | .72 | No |
| Working unit | Feeling uneasy in nursing practice after the enactment of the Turkish Penal Code | 11.008 | 10 | .357 | .118 | 2 (11.1%) | 2.41 | No |
| Daily number of patients cared for | Concern about making mistakes in nursing care | 2.481 | 6 | .871 | .056 | 0 (0.0%) | 9.34 | No |
| Daily number of patients cared for | Administering unnecessary medications to protect oneself from malpractice claims | 8.233 | 6 | .222 | .102 | 2 (16.7%) | 2.74 | No |
| Daily number of patients cared for | Avoiding patients perceived as more likely to file a lawsuit | 6.062 | 6 | .416 | .088 | 2 (16.7%) | 2.23 | No |
| Daily number of patients cared for | Explaining nursing practices in more detail | 6.205 | 6 | .401 | .089 | 4 (33.3%) | 1.93 | No |
| Daily number of patients cared for | Avoiding practices with a high risk of complications | 9.802 | 6 | .133 | .112 | 0 (0.0%) | 5.28 | No |
| Daily number of patients cared for | Keeping medical records in more detail | 1.697 | 6 | .945 | .046 | 8 (66.7%) | 1.02 | No |
| Daily number of patients cared for | Perceived risk of facing a malpractice claim under current working conditions | 5.499 | 6 | .482 | .084 | 0 (0.0%) | 9.14 | No |
| Daily number of patients cared for | Spending more time with patients to protect oneself from malpractice claims | 9.105 | 6 | .168 | .107 | 2 (16.7%) | 2.94 | No |
| Daily number of patients cared for | Paying more attention to informed consent forms | 7.207 | 6 | .302 | .096 | 4 (33.3%) | 1.52 | No |
| Daily number of patients cared for | Feeling uneasy in nursing practice after the enactment of the Turkish Penal Code | 1.297 | 6 | .972 | .041 | 0 (0.0%) | 5.08 | No |
| Previous awareness of defensive medicine | Concern about making mistakes in nursing care | 7.041 | 2 | **.030** | .134 | 0 (0.0%) | 32.69 | **Yes** |
| Previous awareness of defensive medicine | Administering unnecessary medications to protect oneself from malpractice claims | 1.336 | 2 | .513 | .058 | 0 (0.0%) | 9.59 | No |
| Previous awareness of defensive medicine | Avoiding patients perceived as more likely to file a lawsuit | .152 | 2 | .927 | .020 | 0 (0.0%) | 7.82 | No |
| Previous awareness of defensive medicine | Explaining nursing practices in more detail | 3.671 | 2 | .159 | .097 | 0 (0.0%) | 6.75 | No |
| Previous awareness of defensive medicine | Avoiding practices with a high risk of complications | 7.636 | 2 | **.022** | .139 | 0 (0.0%) | 18.48 | **Yes** |
| Previous awareness of defensive medicine | Keeping medical records in more detail | 3.211 | 2 | .201 | .090 | 2 (33.3%) | 3.55 | No |
| Previous awareness of defensive medicine | Perceived risk of facing a malpractice claim under current working conditions | .517 | 2 | .772 | .036 | 0 (0.0%) | 31.98 | No |
| Previous awareness of defensive medicine | Spending more time with patients to protect oneself from malpractice claims | 7.708 | 2 | **.021** | .140 | 0 (0.0%) | 10.30 | **Yes** |
| Previous awareness of defensive medicine | Paying more attention to informed consent forms | .768 | 2 | .681 | .044 | 0 (0.0%) | 5.33 | No |
| Previous awareness of defensive medicine | Feeling uneasy in nursing practice after the enactment of the Turkish Penal Code | 1.793 | 2 | .408 | .067 | 0 (0.0%) | 17.77 | No |
| Self-reported sufficient knowledge about defensive medicine | Concern about making mistakes in nursing care | 3.446 | 2 | .179 | .094 | 0 (0.0%) | 17.05 | No |
| Self-reported sufficient knowledge about defensive medicine | Administering unnecessary medications to protect oneself from malpractice claims | 1.244 | 2 | .537 | .056 | 0 (0.0%) | 5.00 | No |
| Self-reported sufficient knowledge about defensive medicine | Avoiding patients perceived as more likely to file a lawsuit | 1.582 | 2 | .453 | .063 | 1 (16.7%) | 4.08 | No |
| Self-reported sufficient knowledge about defensive medicine | Explaining nursing practices in more detail | 1.331 | 2 | .514 | .058 | 2 (33.3%) | 3.52 | No |
| Self-reported sufficient knowledge about defensive medicine | Avoiding practices with a high risk of complications | 1.391 | 2 | .499 | .059 | 0 (0.0%) | 9.63 | No |
| Self-reported sufficient knowledge about defensive medicine | Keeping medical records in more detail | .530 | 2 | .767 | .037 | 2 (33.3%) | 1.85 | No |
| Self-reported sufficient knowledge about defensive medicine | Perceived risk of facing a malpractice claim under current working conditions | 5.075 | 2 | .079 | .113 | 0 (0.0%) | 16.68 | No |
| Self-reported sufficient knowledge about defensive medicine | Spending more time with patients to protect oneself from malpractice claims | .471 | 2 | .790 | .035 | 0 (0.0%) | 5.37 | No |
| Self-reported sufficient knowledge about defensive medicine | Paying more attention to informed consent forms | .741 | 2 | .690 | .043 | 2 (33.3%) | 2.78 | No |
| Self-reported sufficient knowledge about defensive medicine | Feeling uneasy in nursing practice after the enactment of the Turkish Penal Code | .641 | 2 | .726 | .040 | 0 (0.0%) | 9.26 | No |
| Belief that malpractice lawsuits affect nursing performance | Concern about making mistakes in nursing care | 9.078 | 2 | **.011** | .152 | 0 (0.0%) | 19.15 | **Yes** |
| Belief that malpractice lawsuits affect nursing performance | Administering unnecessary medications to protect oneself from malpractice claims | .633 | 2 | .729 | .040 | 0 (0.0%) | 5.62 | No |
| Belief that malpractice lawsuits affect nursing performance | Avoiding patients perceived as more likely to file a lawsuit | 1.312 | 2 | .519 | .058 | 1 (16.7%) | 4.58 | No |
| Belief that malpractice lawsuits affect nursing performance | Explaining nursing practices in more detail | 8.293 | 2 | **.016** | .145 | 2 (33.3%) | 3.95 | **Yes** |
| Belief that malpractice lawsuits affect nursing performance | Avoiding practices with a high risk of complications | 1.224 | 2 | .542 | .056 | 0 (0.0%) | 10.82 | No |
| Belief that malpractice lawsuits affect nursing performance | Keeping medical records in more detail | 18.442 | 2 | **<.001** | .216 | 2 (33.3%) | 2.08 | **Yes** |
| Belief that malpractice lawsuits affect nursing performance | Perceived risk of facing a malpractice claim under current working conditions | .343 | 2 | .842 | .030 | 0 (0.0%) | 18.73 | No |
| Belief that malpractice lawsuits affect nursing performance | Spending more time with patients to protect oneself from malpractice claims | 6.027 | 2 | **.049** | .124 | 0 (0.0%) | 6.04 | **Yes** |
| Belief that malpractice lawsuits affect nursing performance | Paying more attention to informed consent forms | 1.485 | 2 | .476 | .061 | 1 (16.7%) | 3.12 | No |
| Belief that malpractice lawsuits affect nursing performance | Feeling uneasy in nursing practice after the enactment of the Turkish Penal Code | 2.757 | 2 | .252 | .084 | 0 (0.0%) | 10.41 | No |
| Knowledge of malpractice-related provisions of the Turkish Penal Code | Concern about making mistakes in nursing care | .702 | 2 | .704 | .042 | 0 (0.0%) | 19.15 | No |
| Knowledge of malpractice-related provisions of the Turkish Penal Code | Administering unnecessary medications to protect oneself from malpractice claims | 2.822 | 2 | .244 | .085 | 0 (0.0%) | 5.62 | No |
| Knowledge of malpractice-related provisions of the Turkish Penal Code | Avoiding patients perceived as more likely to file a lawsuit | .815 | 2 | .665 | .045 | 1 (16.7%) | 4.58 | No |
| Knowledge of malpractice-related provisions of the Turkish Penal Code | Explaining nursing practices in more detail | 1.424 | 2 | .491 | .060 | 2 (33.3%) | 3.95 | No |
| Knowledge of malpractice-related provisions of the Turkish Penal Code | Avoiding practices with a high risk of complications | .289 | 2 | .866 | .027 | 0 (0.0%) | 10.82 | No |
| Knowledge of malpractice-related provisions of the Turkish Penal Code | Keeping medical records in more detail | 3.870 | 2 | .144 | .099 | 2 (33.3%) | 2.08 | No |
| Knowledge of malpractice-related provisions of the Turkish Penal Code | Perceived risk of facing a malpractice claim under current working conditions | .574 | 2 | .751 | .038 | 0 (0.0%) | 18.73 | No |
| Knowledge of malpractice-related provisions of the Turkish Penal Code | Spending more time with patients to protect oneself from malpractice claims | 1.074 | 2 | .585 | .052 | 0 (0.0%) | 6.04 | No |
| Knowledge of malpractice-related provisions of the Turkish Penal Code | Paying more attention to informed consent forms | 1.485 | 2 | .476 | .061 | 1 (16.7%) | 3.12 | No |
| Knowledge of malpractice-related provisions of the Turkish Penal Code | Feeling uneasy in nursing practice after the enactment of the Turkish Penal Code | 2.504 | 2 | .286 | .080 | 0 (0.0%) | 10.41 | No |
| Having read the Patients’ Rights Regulation | Concern about making mistakes in nursing care | 10.600 | 2 | **.005** | .164 | 0 (0.0%) | 17.28 | **Yes** |
| Having read the Patients’ Rights Regulation | Administering unnecessary medications to protect oneself from malpractice claims | 3.146 | 2 | .207 | .089 | 0 (0.0%) | 5.07 | No |
| Having read the Patients’ Rights Regulation | Avoiding patients perceived as more likely to file a lawsuit | 2.681 | 2 | .262 | .082 | 1 (16.7%) | 4.13 | No |
| Having read the Patients’ Rights Regulation | Explaining nursing practices in more detail | 1.170 | 2 | .557 | .054 | 2 (33.3%) | 3.57 | No |
| Having read the Patients’ Rights Regulation | Avoiding practices with a high risk of complications | 1.919 | 2 | .383 | .070 | 0 (0.0%) | 9.77 | No |
| Having read the Patients’ Rights Regulation | Keeping medical records in more detail | 1.684 | 2 | .431 | .065 | 2 (33.3%) | 1.88 | No |
| Having read the Patients’ Rights Regulation | Perceived risk of facing a malpractice claim under current working conditions | 3.298 | 2 | .192 | .091 | 0 (0.0%) | 16.90 | No |
| Having read the Patients’ Rights Regulation | Spending more time with patients to protect oneself from malpractice claims | 1.737 | 2 | .420 | .066 | 0 (0.0%) | 5.45 | No |
| Having read the Patients’ Rights Regulation | Paying more attention to informed consent forms | 11.272 | 2 | **.004** | .169 | 2 (33.3%) | 2.82 | **Yes** |
| Having read the Patients’ Rights Regulation | Feeling uneasy in nursing practice after the enactment of the Turkish Penal Code | 8.594 | 2 | **.014** | .148 | 0 (0.0%) | 9.39 | **Yes** |
| Institutional informed consent status | Concern about making mistakes in nursing care | 2.448 | 4 | .654 | .056 | 0 (0.0%) | 10.04 | No |
| Institutional informed consent status | Administering unnecessary medications to protect oneself from malpractice claims | 4.206 | 4 | .379 | .073 | 4 (44.4%) | 2.95 | No |
| Institutional informed consent status | Avoiding patients perceived as more likely to file a lawsuit | 4.858 | 4 | .302 | .079 | 4 (44.4%) | 2.40 | No |
| Institutional informed consent status | Explaining nursing practices in more detail | 1.922 | 4 | .750 | .049 | 4 (44.4%) | 2.07 | No |
| Institutional informed consent status | Avoiding practices with a high risk of complications | 1.845 | 4 | .764 | .048 | 0 (0.0%) | 5.68 | No |
| Institutional informed consent status | Keeping medical records in more detail | 2.508 | 4 | .643 | .056 | 4 (44.4%) | 1.09 | No |
| Institutional informed consent status | Perceived risk of facing a malpractice claim under current working conditions | 4.484 | 4 | .344 | .075 | 0 (0.0%) | 9.82 | No |
| Institutional informed consent status | Spending more time with patients to protect oneself from malpractice claims | 3.186 | 4 | .527 | .064 | 4 (44.4%) | 3.16 | No |
| Institutional informed consent status | Paying more attention to informed consent forms | 7.626 | 4 | .106 | .098 | 4 (44.4%) | 1.64 | No |
| Institutional informed consent status | Feeling uneasy in nursing practice after the enactment of the Turkish Penal Code | 6.716 | 4 | .152 | .092 | 0 (0.0%) | 5.46 | No |

***Note.*** *Pearson’s chi-square tests were used to examine associations between categorical participant characteristics, malpractice-related awareness variables and individual items related to possible defensive nursing practices. Five-point Likert-type responses were collapsed into three categories for these analyses: never/rarely, sometimes and frequently/always. χ², chi-square statistic; df, degrees of freedom; p, probability value; Cramér’s V, effect size for the strength of association. Expected cell counts were checked for each analysis; the number and percentage of cells with expected counts below 5 and the minimum expected count are reported to support transparent interpretation of chi-square assumptions. Previous malpractice lawsuit experience was not included in the association analyses because only seven participants reported such experience, resulting in sparse data. Statistically significant associations are indicated in the final column.*
