# Supplementary material for: Defensive Nursing Practices Among Nurses Working in Private Hospitals: A Cross‐Sectional Study From Türkiye
Source: J Nurs Manag. 2026 Nov 6;2026:3933585. doi: 10.1155/jonm/3933585 (PMC13743797; doi:10.1155/jonm/3933585)
Supplement: Supplementary file 2 [file JONM-2026-3933585-s002.docx]

**STROBE Statement—Checklist of items for cross-sectional studies**

**Manuscript title:** Defensive Medicine Practices Among Nurses Working in Private Hospitals: A Cross-Sectional Study from İstanbul, Türkiye

| **Item No** | **Recommendation** | **Location in manuscript** |
| --- | --- | --- |
| 1(a) | Indicate the study design with a commonly used term in the title or abstract. | Title; Abstract—Methods |
| 1(b) | Provide an informative and balanced summary of what was done and what was found. | Abstract |
| 2 | Explain the scientific background and rationale for the investigation. | Introduction |
| 3 | State specific objectives, including any prespecified hypotheses. | Introduction, final paragraph |
| 4 | Present key elements of study design early in the paper. | Methods—Study design |
| 5 | Describe the setting, locations and relevant dates, including periods of recruitment, exposure, follow-up and data collection. | Methods—Setting and participants; Methods—Data collection |
| 6(a) | Give the eligibility criteria, and the sources and methods of selection of participants. | Methods—Setting and participants |
| 7 | Clearly define all outcomes, exposures, predictors, potential confounders and effect modifiers. | Methods—Data collection instrument; Methods—Defensive medicine practice items |
| 8 | Give sources of data and details of methods of assessment for each variable. | Methods—Data collection instrument; Methods—Defensive medicine practice items |
| 9 | Describe any efforts to address potential sources of bias. | Methods—Data collection; Ethical considerations; Discussion—Limitations |
| 10 | Explain how the study size was arrived at. | Methods—Setting and participants |
| 11 | Explain how quantitative variables were handled in the analyses. | Methods—Statistical analysis |
| 12(a) | Describe all statistical methods, including those used to control for confounding. | Methods—Statistical analysis |
| 12(b) | Describe any methods used to examine subgroups and interactions. | Not applicable; no subgroup or interaction analyses were performed beyond item-level chi-square analyses |
| 12(c) | Explain how missing data were addressed. | Methods—Statistical analysis |
| 12(d) | Describe analytical methods taking account of sampling strategy. | Not applicable; no complex sampling design was used |
| 12(e) | Describe any sensitivity analyses. | Not applicable; sensitivity analyses were not performed |
| 13(a) | Report numbers of individuals at each stage of the study. | Results—Participant characteristics |
| 13(b) | Give reasons for non-participation at each stage. | Not applicable / not available |
| 13(c) | Consider use of a flow diagram. | Not applicable |
| 14(a) | Give characteristics of study participants. | Results—Participant characteristics; Table 1 |
| 14(b) | Indicate number of participants with missing data for each variable of interest. | Methods—Statistical analysis; no missing data were present in the variables included in the final analyses |
| 15 | Report numbers of outcome events or summary measures. | Results—Defensive medicine practice items; Tables 2 and 3 |
| 16(a) | Give unadjusted estimates and, if applicable, confounder-adjusted estimates and their precision. | Results—Factors associated with defensive medicine practice items; Table 4 |
| 16(b) | Report category boundaries when continuous variables were categorized. | Table 1; Methods—Statistical analysis |
| 16(c) | If relevant, consider translating estimates of relative risk into absolute risk. | Not applicable |
| 17 | Report other analyses done, such as analyses of subgroups and interactions, and sensitivity analyses. | Not applicable |
| 18 | Summarise key results with reference to study objectives. | Discussion, first paragraph |
| 19 | Discuss limitations, taking into account sources of potential bias or imprecision. | Discussion—Limitations |
| 20 | Give a cautious overall interpretation of results considering objectives, limitations, multiplicity of analyses, results from similar studies and other relevant evidence. | Discussion |
| 21 | Discuss the generalisability of the study results. | Discussion—Limitations |
| 22 | Give the source of funding and the role of funders. | Declarations—Funding |
